# Narrative exposure therapy: an evidence-based treatment for multiple and complex trauma

**DOI:** 10.3402/ejpt.v5.26522

**Published:** 2014-12-09

**Authors:** Ruud A. Jongedijk

**Affiliations:** Foundation Centrum '45 and Arq Psychotrauma Expert Group, Diemen, The Netherlands

**Keywords:** NET, narratives, exposure, refugees, complex trauma, PTSD, evidence-based treatment

## Abstract

Narrative exposure therapy (NET) is a recently developed, short-term treatment for patients with a posttraumatic stress disorder (PTSD) as a result of multiple trauma. NET can be applied very successfully in patients with complex trauma complaints (Jongedijk, 2014; Schauer, Neuner, & Elbert, 2011).

An important feature of NET is that trauma processing is never an isolated event but is always embedded in the context of a traumatic event and in the life history as a whole. At the start, the lifeline is laid. The lifeline is made up of a rope, with flowers (happy events), stones (traumatic events), sometimes candles (grief), or recently also sticks for aggressive acts (NET for offenders; see Stenmark, Cuneyt Guzey, Elbert, & Holen, 2014). These symbols are laid down along the rope, in chronological order. Subsequently, in the subsequent therapy sessions the lifeline is processed in chronological order, giving attention to all the important events a person has experienced in his or her life, both the adverse as well as the pleasurable ones. The narration ends with a written testimony.

To date, there is good evidence NET is effective in the treatment of PTSD patients, with support from 18 RCTs (*N*=950). For culturally diverse populations, NET is recommended as the most evidence-based trauma treatment, besides culturally adapted CBT. NET has been investigated in different populations in Africa, Europe, and Asia. In Asia, research has been carried out in Sri Lanka as well as in China. In China, NET was conducted and investigated with survivors of the Sichuan earthquake (Zang, Hunt, & Cox, 2013, 2014). NET is understandable, even appealing and also supportive for patients with multiple trauma. In this presentation, the treatment principles and the practice of NET will be explained.
